# Database of human well-being and eco-sustainability under planetary pressures of the Belt and Road 1990–2018

**DOI:** 10.1038/s41597-023-02231-x

**Published:** 2023-05-20

**Authors:** Dawei Zhang, Zicong Guo, Yigong Gao, Xiaxia Niu, Jiayi He, Xinyi Liu, Xinyi Fu, Hui Xu

**Affiliations:** 1 State Key Laboratory of Herbage Improvement and Grassland Agro-ecosystems, College of Ecology, Lanzhou University, Lanzhou, 730000 China; 2 School of Economics, Lanzhou University, Lanzhou, 730000 China

**Keywords:** Sustainability, Environmental impact

## Abstract

The Belt and Road (B&R) Initiative is considered as closely aligned with the UN’s Sustainable Development Goals by 2030 and could have a huge global impact. Its sustainable development issues have attracted worldwide attention. However, both the existing research and data accumulation on this aspect are seriously insufficient. Starting from the logic of the ultimate goal of sustainable development (namely within the ecological limitations, maximizing human well-being with minimum ecological consumption and minimizing the planetary pressures with maximum resource utilization efficiency), we have constructed a comprehensive evaluation method on sustainable development, namely the Consumption-Pressure-Output-Efficiency method in our previous study. Based on it, we provide a database with five datasets, which includes four core datasets (ecological consumption, planetary pressures, human well-being outputs and ecological well-being output efficiency) and a related dataset (biocapacity, ecological surplus/deficit, population), covering 61 B&R countries, B&R regional average and global average from 1990 to 2018. It can be used for further comprehensive research on sustainable development under planetary pressures and others of B&R.

## Background & Summary

The Belt and Road Initiative (BRI, also known as One Belt, One Road or B&R), which was proposed by China in 2013, is considered closely aligned with the UN’s Sustainable Development Goals (SDGs) by 2030 and could have a huge global impact^1,2^. It is a brand-new and grand initiative that has attracted worldwide attention^3,4^. Due to the main portion of the B&R being located in arid or semi-arid areas or sub-humid ecologically fragile zones, with the sensitive, fragile and weak self-recovery ability of the eco-environment, its response to intensified global climate change and human activities will become more sensitive^5,6^. Thus, its sustainable development issues have attracted worldwide attention^2,7^.

Sustainable development was defined by the United Nations World Commission on Environment and Development as “development that meets the needs of the present without compromising the ability of future generations to meet their own needs” in 1987^8^. However, the discussion on ‘sustainable’ has always existed, the concept of ‘sustainable’ is still controversial, and different research groups have assigned different concepts and meanings to ‘sustainable’^9^. With a deeper understanding about it, the ultimate goal of sustainable development is, within the ecological limitations, to expand human’s developmental potential to maximize human well-being with the smallest ecological consumption, to maximize the efficiency of resource conversion while easing the planetary pressures^10–12^. It makes sense to define the subject of sustainability as a relationship among economic, social, and ecological systems, including economic society in the drivers of ecosystem change, and apply it to a geographical scale^13^. Thus, the evaluation of sustainable development needs to consider both ‘sustainable’ and ‘development’, and economic, social and environmental dimensions, to meet both ‘sustainability’ i.e., that human activities must not exceed the bio-capacity, and ‘development’ i.e., the continuous improvement of human well-being^12,14,15^, and ultimately realize sustainable development for both humans and nature.

For the evaluation of sustainable development, many methods have been developed, and can be generally divided into two categories, namely index system and indicators method^16^. The index system method is an index set comprising almost all basic indicators in the major areas of sustainable development, which aims to evaluate the extent of the ecological impact of human activities. Such as the Pressure-State-Response model^17^, Driving-Pressure-State-Impact-Response model^18^, and SDGs^19,20^. The advantage of the index system method is that it is rich in indicators and can cover environmental, economic and social aspects. The other is the indicators method, which usually includes an evaluation indicator and composite indicator. An evaluation indicator is a single index formed by mathematical calculation results of several core basic indexes, such as ecological footprint (EF) method^21^ and human development index (HDI)^22^. Composite indicator is a combination of many single indicators and is a powerful tool for change, innovation and political signals^11^, such as ecological well-being performance (EWP) indicator^23^. The advantage of the indicators method is that the indicators are concise, simple to be operated, easy to be understood and some indicators are already widely recognized and used to do comparisons between countries.

However, there are also some deficiencies in these existing methods. For instance, the index system method, either lacks a unified index selection standard, leading to inconsistent indexes selected by different studies and limiting the comparison between researches, or the index number is too much and complicated, resulting in difficulties in data acquisition and processing and lacking the consideration of interaction and influence relationship among indicators^24,25^. The indicator method, either often does not cover all the dimensions of society, economy and environment (such as single indicators, EF focuses only on the environmental dimension and does not include the economic and social dimension^26,27^, HDI does not consider environmental factors and intergenerational inequality^28,29^, leading to the evaluation results are not comprehensive enough owing to the different emphases of each method^27^), or often covers up certain problems, for it is rarely clear or transparent^11^ (such as the composite indicator, EWP can show the rate of conversion of ecological consumption into human well-being, but cannot directly show the true level of well-being^30,31^). It is clear that we cannot rely on a single index or measure to obtain the comprehensive evaluation result of sustainable development, not to mention in the B&R regions, which lacks uniform available database resources.

**Fig. 1:**
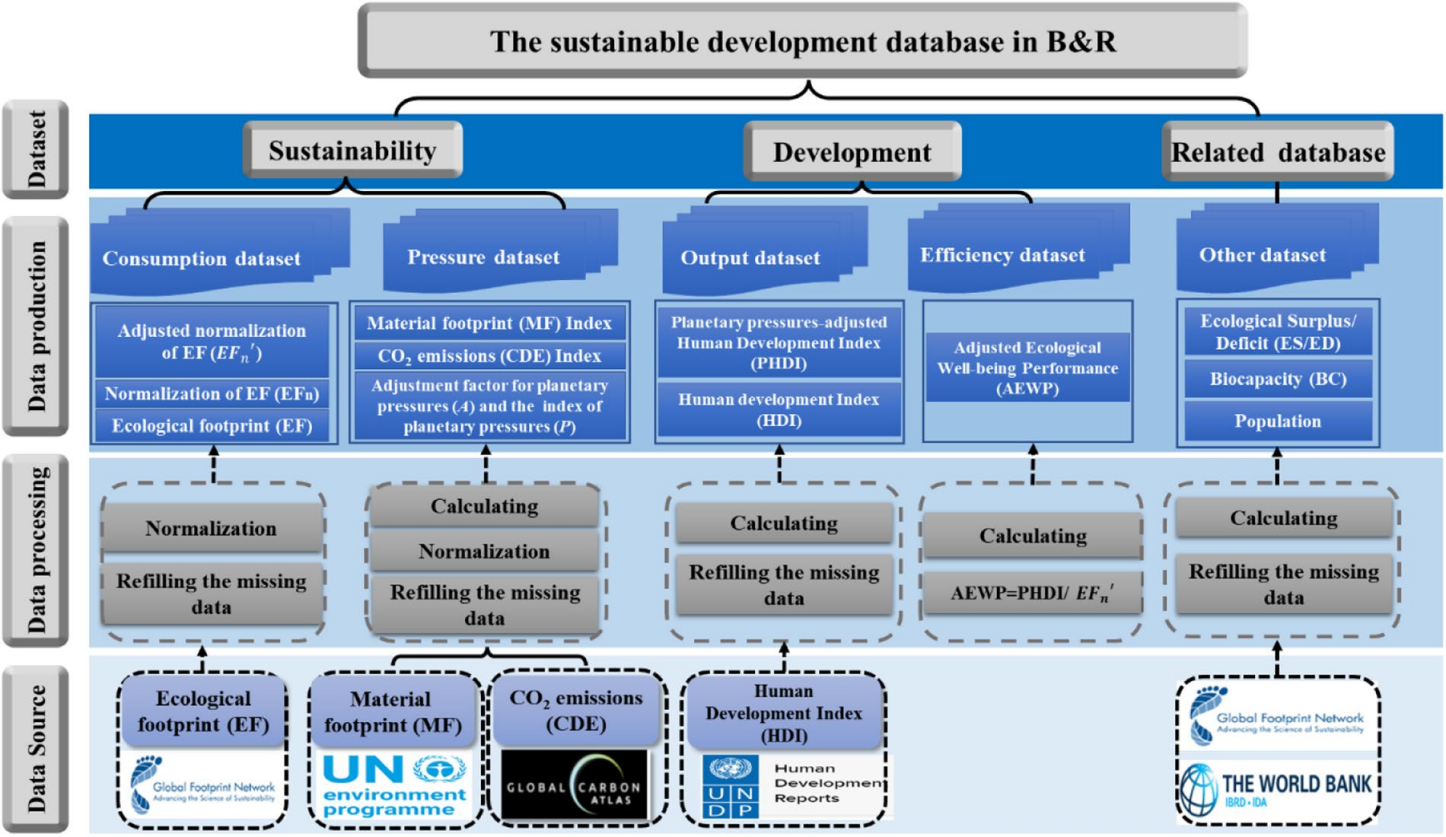
The construction of sustainable development database in B&R.

All these make us go back and review the proposal, evolution and latest progress of the concept of sustainable development mentioned above to see if we can find a better solution to the problem. According to HDR 2020 that reductions in the flows of greenhouse gases and more efficient material use would eventually reflect the outcomes of the broader economic and societal transformation to ease planetary pressures, and carbon dioxide emissions (CDE) per capita and material footprint (MF) per capita from human activity can be used to represent the planetary pressures^11^. Thus, starting from the logic of the ultimate goal of sustainable development, we constructed a comprehensive evaluation method on sustainable development, namely a consumption–pressure–output–efficiency (CPOE) method^16^, and a database, by combining the index system idea and four composite indicators. That is, in the context of ecological limitation [represented by biocapacity (BC)], to respectively measure how much ecological consumption is generated by human development (by EF), how much planetary pressure is caused by ecological consumption [by the index of planetary pressures (*P*), which is obtained from CDE and MF], how much human well-being is produced by ecological consumption under planetary pressures [by planetary pressures–adjusted HDI (PHDI), which extends the HDI by taking good account of environmental factors (CDE per capita, to illustrate the transition of energy away from fossil fuels) and intergenerational inequality (MF per capita, to illustrate the challenges of closing material cycles) to measure human well-being under planetary pressures^11^, and how much the conversion efficiency of ecological consumption into human well-being [by an EWP index adjusted by PHDI (AEWP)]. The framework can determine whether contemporary human economic and social activities in the face of planetary pressures exceed the ecological carrying capacity (determined by comparing EF and BC), which reflects “sustainability”, whether the level of well-being under planetary pressures is increasing (determined by PHDI), which reflects “development”, and finally how efficient the transition to sustainable development is through AEWP. Thus, it formed a logical system and index that covers both sustainability (consumption and pressure) and development (output and efficiency) (Fig. 1), not only combining the advantages of existing evaluation methods, but also making up for their shortcomings, and all data are available by acquisition or calculation.

Finally, we formed a basic database, which contains 29,621 data records for comprehensive evaluation on sustainable development of B&R. It includes not only four core datasets [including consumption (EF), pressure (CDE, MF, *P*), output (HDI, PHDI) and efficiency (AEWP)], but also one related dataset [including bio-capacity (BC), ecological surplus/deficit (ES/ED) and population]. It covers 61 B&R countries, B&R regional average and global average from 1990–2018, and can be used for further research on sustainable development and others of B&R. The abbreviations in this paper are shown in Table 1.

**Table 1:** The abbreviations for some key concepts.

Abbreviations	
BC	Biocapacity
EF	Ecological Footprint
EF_n_	Normalization of EF
$E{F}_{n}^{{\prime} }$	Adjusted normalization of EF, calculated by $E{F}_{n}^{{\prime} }=E{F}_{n}\times 0.99+0.01$, to avoid EF_n_ = 0
ED	Ecological deficit
ES	Ecological surplus
HDI	Human Development Index
PHDI	Planetary pressures–adjusted Human Development Index
*A*	Adjustment factor for planetary pressures
*P*	The index of planetary pressures
EWP	Ecological Well-being Performance
AEWP	Adjusted Ecological Well-being Performance
CDE	Carbon dioxide emissions
MF	Material footprint

## Methods

### Data collection

We selected 65 B&R countries that are mostly mentioned in the literatures^32,33^ as the primary candidates. Among them, due to the EF per capita data of Maldives, and the MF per capita data of Montenegro, Maldives, Palestine, and East Timor being missing, we finally selected the 61 countries as the objects (Table 2). Meanwhile, due to the available data of HDI for each country spans from 1990 to 2019, while that of the EF is from 1961 to 2018, we ultimately determined that the collection period of pressure and output datasets is 1990–2019 (retained the 2019 data is to facilitate the technical validation that follows), and that of consumption and efficiency datasets is 1990–2018.

**Table 2:** Regional classification of 61 B&R countries.

Region	Countries
Central Asia	Kazakhstan, Kyrgyzstan, Tajikistan, Turkmenistan, Uzbekistan
East Asia	China, Mongolia
Europe	Albania, Belarus, Bosnia and Herzegovina, Bulgaria, Croatia, Czech Republic, Estonia, Hungary, Latvia, Lithuania, Macedonia, Moldova, Poland, Romania, Russia, Serbia, Slovakia, Slovenia, Ukraine
South Asia	Afghanistan, Bangladesh, Bhutan, India, Nepal, Pakistan, Sri Lanka
Southeast Asia	Brunei, Cambodia, Indonesia, Laos, Malaysia, Myanmar, Philippines, Singapore, Thailand, Viet Nam
West Asia & Africa	Armenia, Azerbaijan, Bahrain, Egypt, Georgia, Iran, Iraq, Israel, Jordan, Kuwait, Lebanon, Oman, Qatar, Saudi Arabia, Syria, Turkey, United Arab Emirates, Yemen

The EF and BC per capita data are from the Global Footprint Network website (https://data.footprintnetwork.org/). The CDE per capita and MF per capita data are from the Global Carbon Project (http://www.globalcarbonatlas.org/en/CO2-emissions) and United Nations Environment Programme (UNEP) (https://wesr.unep.org/downloader). The HDI data are from the United Nations Development Programme (UNDP) (https://hdr.undp.org/en/data), while the population data is from the World Bank (https://data.worldbank.org). These official websites will also revise, calibrate and update the data from time to time to ensure the data is timely and effective. What we downloaded is the latest data from each official website. Thus, the main basic data are authoritative, reliable and available. Since most data provided on the websites are per capita data for each country, in order to obtain the data of the B&R regions, we conducted a data inversion, which is to multiply the per capita data of each country by the number of population in the current year, then add it up and divide by the total population of the region, rather than simply aggregate and average the data for each country. Meanwhile, to improve the accuracy of the assessment, interpolation was used to complete the missing years for some countries to make the data more complete^34^.

### Pre-processing of the data

Refill the missing data in the datasets. The MF per capita, EF per capita and HDI of some countries have not been fully counted, and need to be supplemented. For example, for some missing HDI data of 10 countries, we first tried to fill in the missing data by searching the annual Human Development Reports (HDRs), and then used the TREND function in EXCEL to interpolate and fill in the remaining data that could not be obtained. The data supplemented follows the principle that the missing data in the original database for a given year was last counted data in the HDR. We also verified the reliability of the data filled by the TREND function in the technical verification section. All the supplemented data are marked on the EXCEL sheet we provided and marked the data storage address.

Normalized the EF per capita. As an important tool for measuring ecological consumption from the sources and sinks of consumption^35,36^, EF can define and measure the impact of human activities on natural ecosystems by calculating the supply and demand of natural capital of different land-use types. However, EF is a dimensional value, which needs to be normalized to form an efficiency dataset. We normalize the EF by referring to the natural logarithm function^30^. The formula is as follows:

1 $$E{F}_{n}=\frac{ln\left(EF\right)-{\rm{ln}}\left(E{F}_{min}\right)}{ln\left(E{F}_{max}\right)-{\rm{ln}}\left(E{F}_{min}\right)}$$

Where *EF*_*max*_ should be the largest value and *EF*_*min*_ should be the smallest value which were observed in 61 countries since 1990. From the global EF network, during 1990–2018, in B&R countries, the maximum value is 16.51 gha per capita, observed in Qatar in 2007, and the minimum is 0.46 gha per capita in Bangladesh in 1994. In worldwide, the maximum value is 17.73 gha per capita in Luxembourg in 2003, and the minimum is 0.43 gha per capita in East Timor in 2011.

Measurement of the PHDI. Following the HDR 2020 prepared by the UNDP, the HDI was adjusted by considering two indexes to reflect the pressures that humans have exerted on the planet during development. That is, the HDI is adjusted by the planetary pressures adjustment factor *A*, which is formed from the arithmetic average of the CDE index (using CDE per capita, from human activities—primarily combustion and the use of coal, oil and gas in industrial processes—to illustrate the challenges of moving away from fossil fuel energy) and the MF index (using the material footprint per capita—a country’s attribution between global material extraction and domestic final demand—to illustrate the challenge of closing the material cycle)^11^. Accordingly, the index of planetary pressures (*P*) is 1 − *A*. To verify the accuracy, we also compared the PHDI dataset for 61 B&R countries in 2019 with that of HDR 2020 compiled by the UNDP in the technical verification section. The formulas are as follows^11^:

2 $${A}_{j}index=\frac{maximu{m}_{j}-observed\;valu{e}_{j}}{maximu{m}_{j}-minimu{m}_{j}}$$

3 $$A=\frac{CDE\;index+MF\;index}{2}$$

4 $$P=1-A$$

5 $$PHDI=HDI\times A$$

Where j = 1, 2 represents the CDE (production) index and MF index, respectively; *A* is the adjustment factor for planetary pressures, *P* represents the index of planetary pressures, and PHDI represents the planetary pressures-adjusted HDI. Following the 2020 PHDI Technical Note, the ${A}_{minimu{m}_{j}}$ is set to zero as an ideal scenario with no planetary pressures^11^. Since this study provides a database for the B&R regions and the global, there are two maximum values. By observing the B&R regions and the worldwide, it was found that the maximum values of carbon dioxide emissions per capita and material footprint per capita are consistent respectively. The ${A}_{maximu{m}_{j}}$ correspond to the maximum value observed historically for all 61 B&R countries and the world as a whole from 1990–2019 respectively. For CDE per capita, the maximum value is 68.72 tonnes, observed in Qatar in 1997. For MF per capita, the maximum is 107.42 tonnes in Kuwait in 1996.

Measurement of the AEWP. Similar to eco-efficiency, EWP uses the ratio of HDI and EF to express the conversion rate of ecological consumption into human well-being and can be used as one of important indexes to measure sustainable development^23^. Here, we have formed a new EWP called AEWP, by replacing HDI with PHDI. It not only retains the advantages of EWP, including the social, economic and environmental dimensions of sustainable development, and is applicable to various geographical scales (global, regional, national, and community), but also takes into account the planetary pressures caused by human development. Meanwhile, as the denominator for calculating AEWP (formula 7), the $E{F}_{n}^{{\prime} }$ cannot be 0. To avoid the value of 0, we reprocess the $E{F}_{n}^{{\prime} }$ by EF_n_ × 0.99 + 0.01^16^, by combining and improving the efficacy coefficient method^37^. The formulas are as follows:

6 $$E{F}_{n}^{{\prime} }=E{F}_{n}\times 0.99+0.01$$

7 $$AEWP=\frac{PHDI}{E{F}_{n}^{{\prime} }}$$

By this way, the standardized value is increased by 0.01 overall and will be between 0.01 and 1. Not only keeping the variation range of the value consistent and avoiding the occurrence of extreme value of 0, but also as close as possible to the true value, effectively reducing the impact of extreme values.

## Data Records

The database totally contains 29,621 data records, including 11,154 raw data (37.66% of the total), 230 supplementary data (2.06% of the total raw data) and 18,237 pre-processing data (61.57% of the total). Among them, there are 18,467 new data (including the supplementary data and pre-processing data, 62.34% of the total) that were not available in the public database at all before our database was published. The database was recorded in four core datasets of consumption, pressure, output and efficiency (Table 3), and one related dataset (Table 4), covering both sustainability and development aspects of sustainable development.

**Table 3:** The four core datasets file descriptions.

Dataset types	Data types	Index and Value	Unit	Scale	Period	Data file name	Data description
Consumption dataset	Raw data	Ecological Footprint (EF) per capita	gha	61 countries and the global	1990–2018	Consumption dataset.xlsx	Used to calculate the EF_n_.
	Pre-processing data	Normalizatio of EF (EF_n_)	—	61 countries and the global	1990–2018	Consumption dataset.xlsx	Used to calculate the $E{F}_{n}^{{\prime} }$, EWP and AEWP.
	Pre-processing data	Adjusted normalization of EF ($E{F}_{n}^{{\prime} }$)	—	61 countries and the global	1990–2018	Consumptiondataset.xlsx	Used to calculate the AEWP.
Pressure dataset	Raw data	Carbon dioxide emissions (CDE) per capita	tonnes	61 countries and the global	1990–2019	Pressure dataset.xlsx	Used to calculate the Carbon dioxide emissions (production) index.
	Pre-processing data	Carbon dioxide emissions (CDE) Index	—	61 countries and the global	1990–2019	Pressure dataset.xlsx	Used to calculate the PHDI.
	Raw data	Material footprint (MF) per capita	tonnes	61 countries and the global	1990–2019	Pressure dataset.xlsx	Used to calculate the Material footprint Index.
	Pre-processing data	Material footprint (MF) Index	—	61 countries and the global	1990–2019	Pressure dataset.xlsx	Used to calculate the PHDI.
	Pre-processing data	Adjustment factor for planetary pressures (*A*) and the index of planetary pressures (*P*)	—	61 countries and the global	1990–2019	Pressure dataset.xlsx	Used to calculate the PHDI.
Output dataset	Raw data	Human Development Index (HDI)	—	61 countries and the global	1990–2019	Output dataset.xlsx	Used to calculate the PHDI.
	Pre-processing data	Planetary pressures- adjusted HDI (PHDI)	—	61 countries and the global	1990–2019	Output dataset.xlsx	Used to calculate the AEWP.
Efficiency dataset	Pre-processing data	Adjusted Ecological Well-being Performance (AEWP)	—	61 countries and the global	1990–2018	Efficiency dataset.xlsx	Used to measure how much the conversion efficiencyof ecological consumption into human well-being.
	Pre-processing data	Ecological Well-being Performance (EWP)	—	61 countries and the global	1990–2018	Efficiency dataset.xlsx	Used to compare with AEWP.

**Table 4:** The related dataset file descriptions.

Dataset types	Data types	Index and Value	Unit	Scale	Period	Data file name	Data description
Other dataset	Raw data	Biocapacity (BC) per capita	gha	61 countries and the global	1990–2018	Other dataset.xlsx	Used to measure the theoretical maximum capacity of an ecosystem to meet human ecological consumption needs.
	Pre-processing data	Ecological Surplus (ES)/ Deficit (ED) per capita	gha	61 countries and the global	1990–2018	Other dataset.xlsx	Used to reflect the difference between the biocapacity and ecological footprint of a region or country.
	Raw data	Population, total	—	61 countries and the global	1990–2019	Other dataset.xlsx	Used to calculate the average level of PHDI and EF per capita of the region.

All the raw data have been carefully checked and there are no particularly large errors (Tables 3,4). Even if individual abnormal data appear during the data processing, they are processed based on the raw data and should be acceptable, because the raw data may reflect some real situation of a country at that time, which does not affect the overall data trend and application. For the pre-processing data, we used the common practice of other scholars and studies to process the data in consistent steps and did not process the data separately for specific countries and years. Some explanations of the four datasets and one related dataset are as follows:

- Consumption dataset, including EF per capita, the Normalization of EF, and the adjusted normalization of EF of 61 B&R countries, B&R regional average and global average from 1990–2018. Among the raw data, there are 57 supplementary data. EF is known as “ecological occupation” and biological production area, which is a biological comprehensive indicator^38^, and an indicator of ecological consumption.
- Pressure dataset, including CDE per capita and its index, MF per capita and its index, *A*, and *P* of 61 B&R countries, B&R regional average and global average from 1990–2019. Among the raw data, there are 6 supplementary data, and they are all MF per capita supplementary data. MF is used to measure consumption-based material resources use, the more resources consumed, the greater the environmental stress. According to Human Development Report 2020, *P* was chosen to measure the level of pressure, *A* was an adjustment factor, and *A* is the arithmetic mean of the index of per capita CO_2_ emissions and per capita MF^11^. Thus, both CDE per capita and MF per capita are essential compositions of the planetary pressures.
- Output dataset, including HDI and PHDI of 61 B&R countries, B&R regional average and global average from 1990–2019. Among the raw data, there are 110 supplementary data.
- Efficiency dataset, including AEWP and EWP of 61 B&R countries, B&R regional average and global average from 1990–2018.
- Other dataset, including BC per capita, ES/ED per capita, and population of 61 B&R countries, B&R regional average and global average from 1990–2018/2019. Among the raw data, there are 57 supplementary data and all are BC per capita supplementary data.

All data are stored as xlsx. files (Tables 3,4), and these datasets are open to the public in the Figshare repository (10.6084/m9.figshare.19948007.v9).

## Technical Validation

### Validation of supplementary data

In our data retrieval and collection process, we find that the EF per capita and BC per capita of 21 countries (Russia, Kuwait, Azerbaijan, Georgia, Armenia, Estonia, Lithuania, Slovenia, Macedonia, Serbia, Croatia, Latvia, Bosnia and Herzegovina, Ukraine, Belarus, Moldova, Kazakhstan, Kyrgyzstan, Tajikistan, Turkmenistan, Uzbekistan), HDI of 10 countries (Bhutan, Oman, Lebanon, Azerbaijan, Georgia, Macedonia, Bosnia and Herzegovina, Belarus, Turkmenistan, Uzbekistan), and MF per capita of 3 countries (Russia, Czech Republic, Serbia) have varying degrees of missing data in given years (Table S1). To ensure full coverage and completeness of the data collection, we supplemented all of these missing data.

For the EF per capita, BC per capita, and MF per capita, for the missing data have no other reliable data sources, we directly use the TREND function in EXCEL to supplement them. To validate the validity of the supplementary data, we fitted the supplementary data for each country with their raw data by linear regressions. For EF per capita, the countries with R^2^ ≥ 0.5 is 9 (by 42.86%), with 0.5 > R^2^ > 0.2 is 5 (by 23.81%) (Figure. S1). For BC per capita, the countries with R^2^ ≥ 0.5 is 12 (by 57.14%), with 0.5 > R^2^ > 0.2 is 6 (by 28.57%) (Figure. S2). For MF per capita, the countries with R^2^ ≥ 0.5 is 2 (by 66.67%) (Figure. S3). Although the explanatory power of linear regressions presents a certain difference, the results still show an overall good fit degree, as the missing data needed to be complemented are not too much. For example, for EF per capita and BC per capita, the most supplementary data is 9 in Kuwait but with R^2^ 0.53 and 0.97, 6 in Azerbaijan with R^2^ 0.41 and 0.45, 5 in Ukraine with R^2^ 0.54 and 0.94, and 3 in Macedonia with R^2^ 0.37 and 0.68, all the rests are 2. For MF per capita, the most supplementary data is 3 in Czech with R^2^ 0.58 and 2 in Serbia with R^2^ 0.87. Thus, the supplementary data is valid and can meet the requirements of the research.

**Fig. 2:**
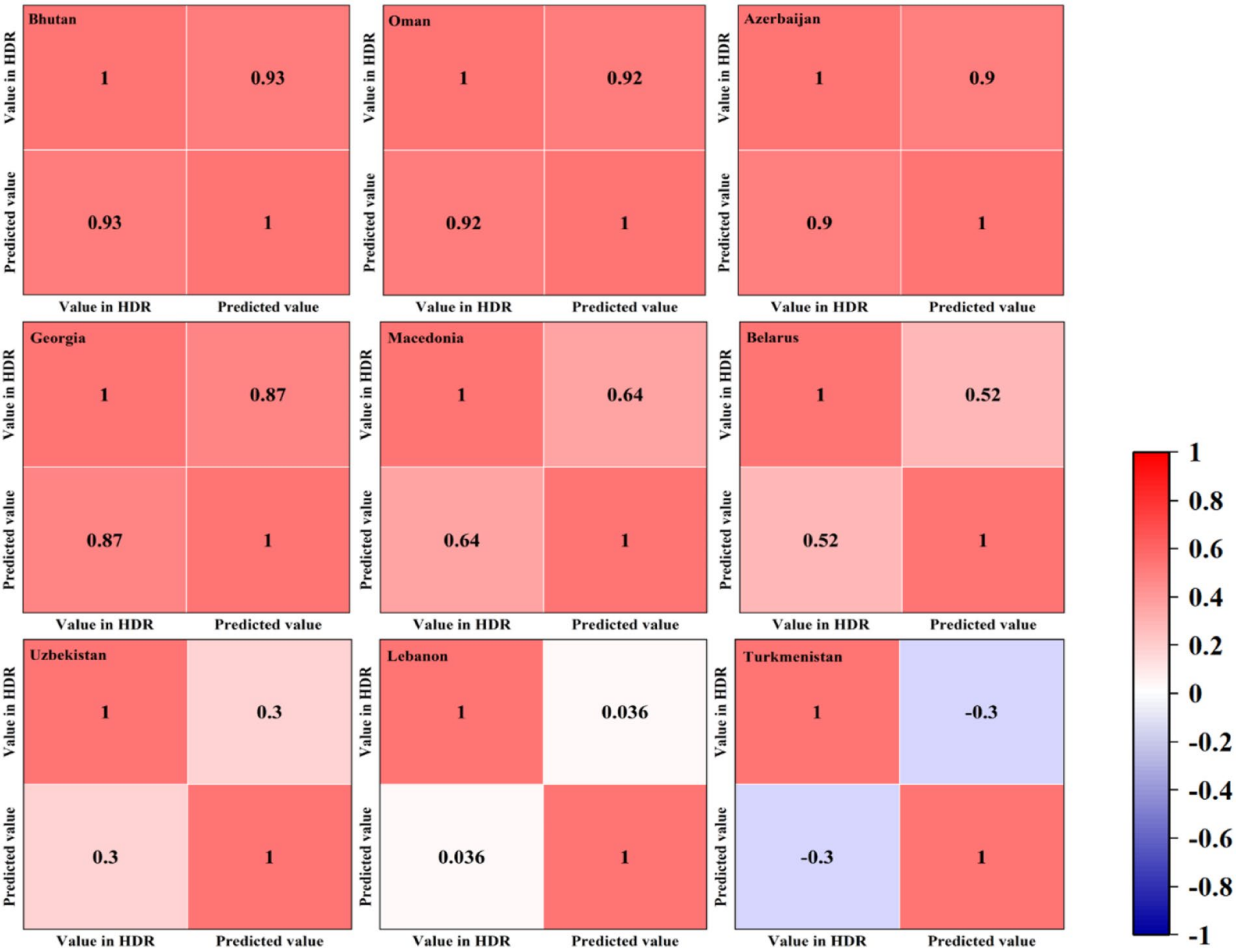
Correlation between HDI supplementary value and HDR’s HDI value.

For HDI, to keep the consistency and coherence of the data, we first tried to fill in the missing data by searching annual HDRs (none of Bosnia and Herzegovina), and the rest were interpolated by the TREND function in EXCEL (including Bosnia and Herzegovina). To validate the validity of the supplementary data, we verified them by two steps. First, we also predicted the supplementary data from HDRs sources by TREND function, and then conducted a Pearson’s correlation analysis between the two. The results show that the correlation between supplementary data and raw data of Bhutan, Oman, Azerbaijan, and Georgia is very strongly positive, that of Macedonia and Belarus is strongly positive, Uzbekistan is weakly positive, Lebanon is very weakly positive, while Turkmenistan is negative (Fig. 2). Though the correlation of the latter two are not strongly positive, due to 12 of 15 missing data of Lebanon, 15 of 20 missing data of Turkmenistan are from HDRs in different years, the data also are valid and can be used. Second, we conducted a fitting analysis of all supplementary data with raw data by linear regressions. The results show that although the goodness of fit varies widely among countries, most of the supplementary data fits well with the raw data, with six countries having a goodness of fit >0.5 (by 60%) (Figure. S4). After re-examination, it is found that the countries with low goodness of fit are mainly due to the large variation (with high dispersion) of the raw data in these countries, and the raw data before and after missing data differ greatly. The deeper reason should be that HDI involves economic and social dimensions, and consists of Life Expectancy, Education Index and GNI index^11^, while these three indexes will change somewhat due to annual revisions. In addition, the annual population count will change slightly due to the revision. But overall, the supplementary data will still be within the reasonable variation range of the raw data. Therefore, the missing data completed by the TREND function can be used in the subsequent calculation process.

### Comparison with existing PHDI estimates

There are two purposes for this comparison. One is to correct some errors in HDR 2020, and the other is to verify the reliability of the acquired data. HDR 2020 provided a calculation method for calculating *A* (aim to obtain the corresponding *P* value, 1-*A* = *P*) and PHDI, but it only calculated *A* and PHDI for the year 2019. Meanwhile, since there is no 2019 updated data of CDE and MF, HDR 2020 adopted the CDE data of 2018 and the MF data of 2017^11^. This may lead to a deviation in PHDI calculation and be hard to accurately measure the true level of human development in each country for the given year.

**Fig. 3:**
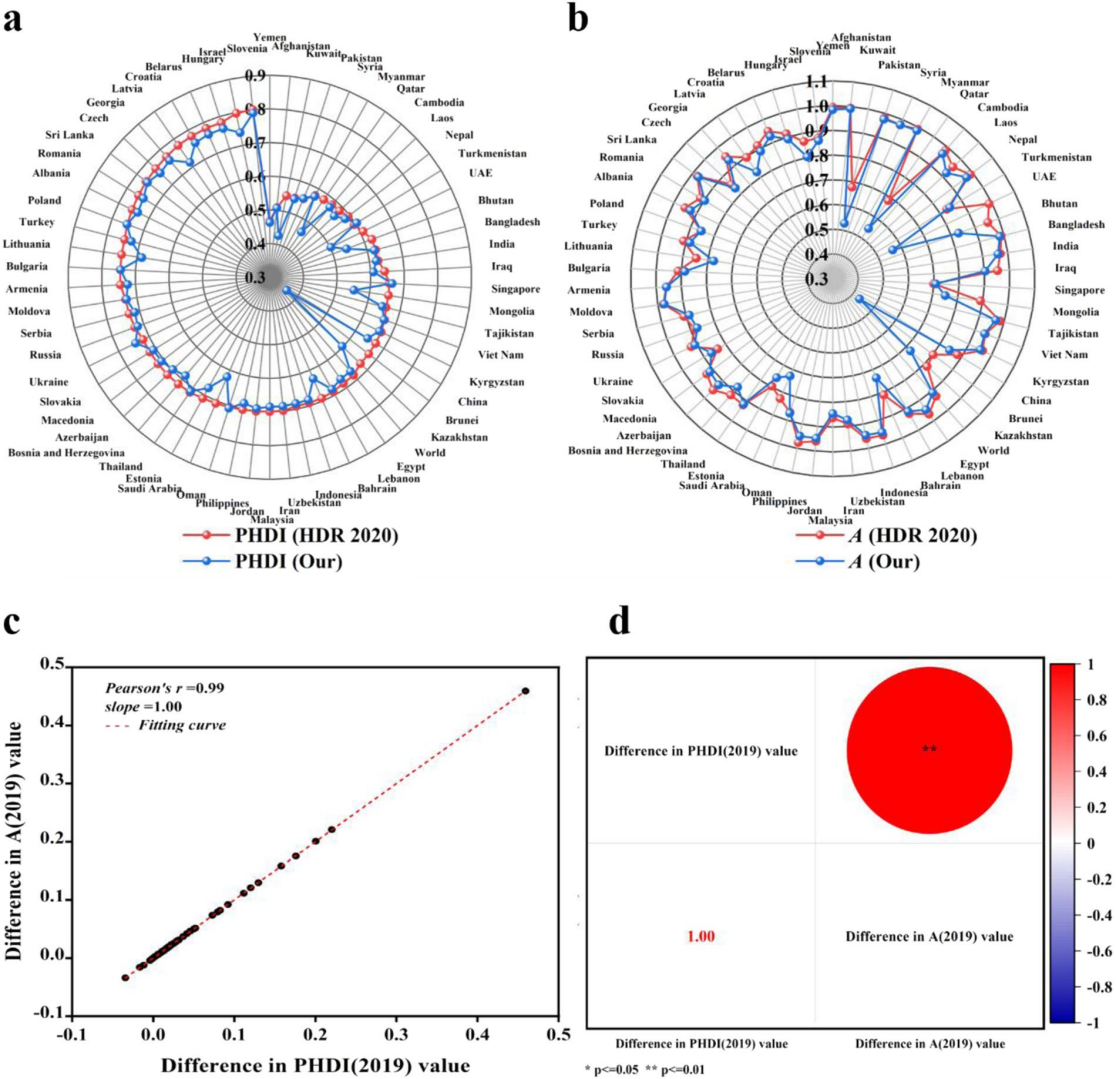
The comparison of PHDI (2019) (**a**) and *A* (2019) (**b**) between HDR 2020 and our results, and the correlations of the difference in PHDI (**c**) and *A* (**d**).

In this study, based on the basic method provided by HDR 2020, we calculated *A* and PHDI for 61 B&R countries from 1990 to 2019 (among them, the data in 2019 is mainly for verification and comparison with the results in HDR 2020), and the updated 2019 CDE data and MF data were used. We took 2019 as an example to compare the results between HDR 2020’s and ours. The results showed that there had been some changes between our revised PHDI and *A* and that of HDR 2020 (Fig. 3a,b). Since PHDI is adjusted by *A* (Eq. 5), the changes of *A* and PHDI should be consistent and should be fit. To verify the reliability and consistency of our results, we fitted the variation differences between HDR 2020 and our revised PHDI and *A*, respectively. It is noteworthy that the difference of PHDI is highly correlated with that of *A*, with a correlation coefficient of 0.99 (Fig. 3c), extremely significant at probability (*p*) value ≤ 0.01 (Fig. 3d). It further states that our data collection and the method we used is consistent with HDR 2020 and the data we obtained is reliable and valid.

### Comparison with existing EWP estimates

**Fig. 4:**
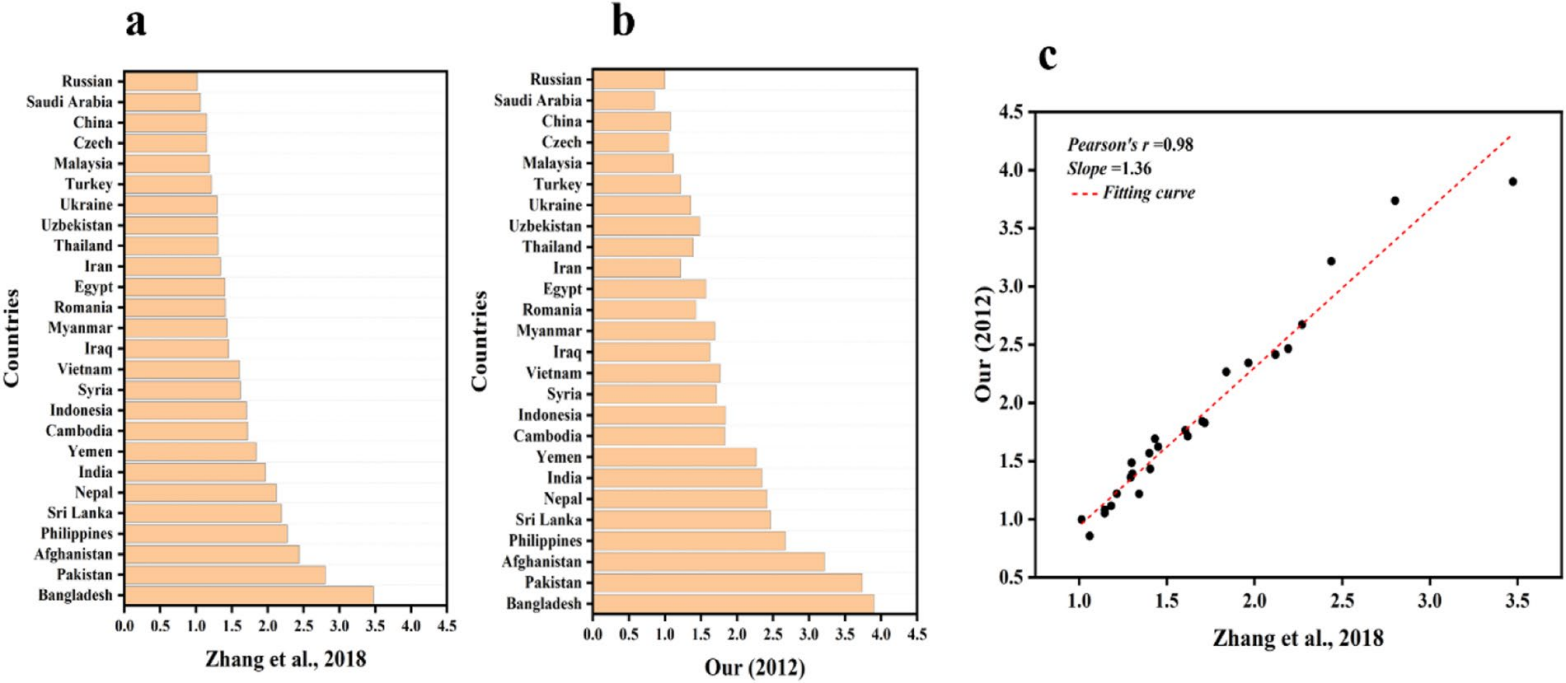
The comparison of values and rankings between EWP (**a**) and AEWP (**b**), and the value correlations between EWP and AEWP in 2012 (**c**).

**Fig. 5:**
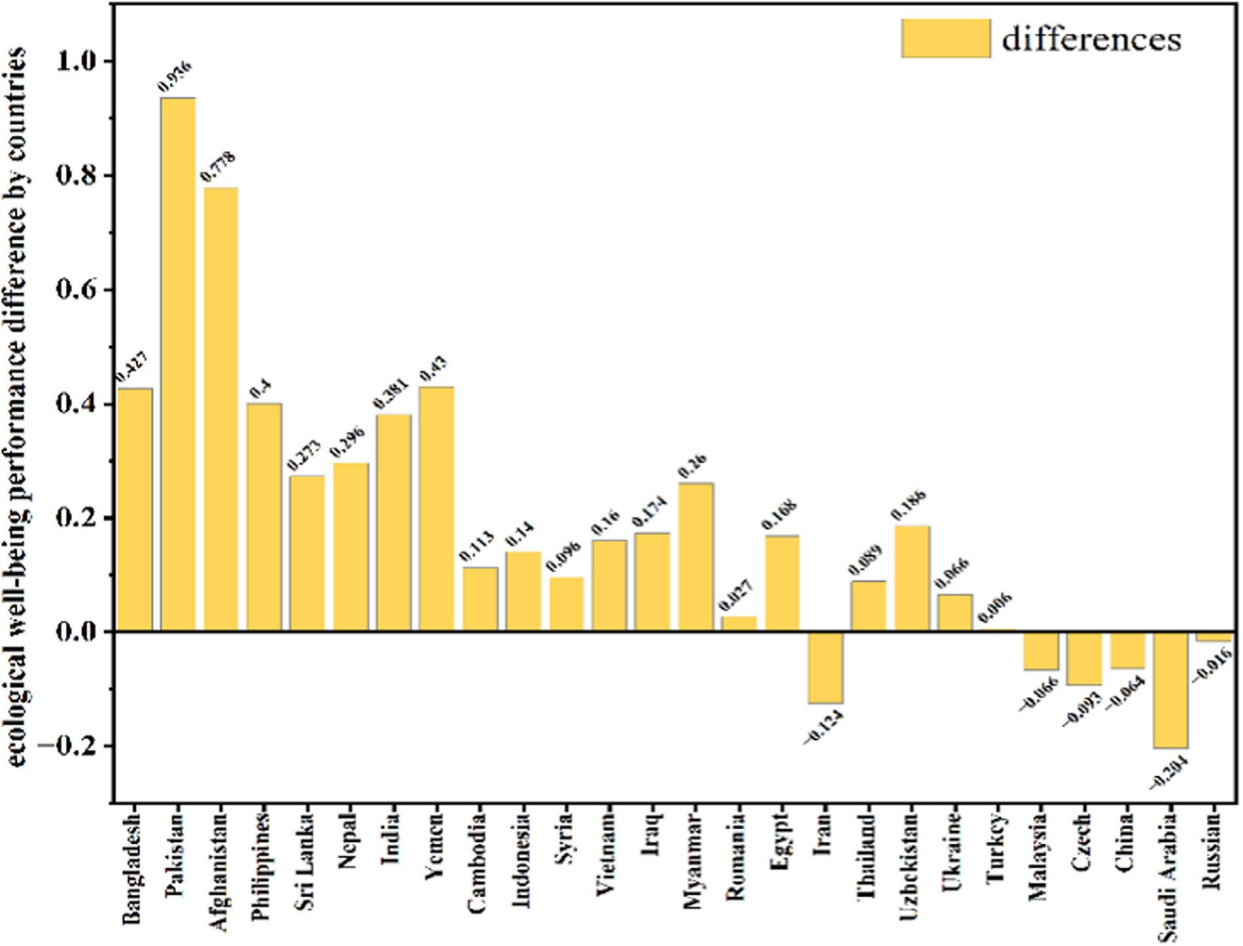
The difference in ecological well-being performance between countries in 2012 for this study (AEWP) and Zhang’s (EWP).

This is to verify the reasonableness and reliability of our revisions to EWPs. On the one hand, with the deepening of understanding, we believe that replacing HDI with PHDI and revising EWP to AEWP should be more scientific and reasonable. On the other hand, although Zhang *et al*. (2018) calculated the EWP, due to the lack of data and other factors, they only calculated and provided one year EWP data of 82 countries in 2012, and did not form a long time series EWP dataset that can be used. In this study, we sifted out the same countries as Zhang’s, and then extracted the 2012 AEWP of those countries from our dataset and to fit them with the 2012 EWP of Zhang’s. Firstly, we calculated the AEWP of 61 B&R countries from 1990 to 2018, using a revised EWP that replaced HDI with PHDI, which is a HDI that taken into account the planetary pressures. Then, the EWP of 82 countries in 2012 was measured by using the ratio of HDI to EF, and each country’s EWP was ranked^30^. There are 26 countries overlapped with B&R. Therefore, we sifted out these 26 common countries and compared their EWP, AEWP values and rankings in 2012. The results showed that the values and rankings of these countries changed to varying degrees, the values of most countries have not changed much, while the rankings of 61.54% of countries had changed (Fig. 4a,b). Meanwhile, the correlation between EWP and AEWP is extremely strong, with a pearson’s *r* = 0.98 (Fig. 4c). Again, the difference between the results of this study and Zhang’s ecological well-being performance varies from country to country. As shown in Fig. 5, EWP estimates are higher than AEWP by more than 0.05 in 18 countries and lower than AEWP by more than 0.05 in 5 countries, accounting for 88.46% of the 26 countries. For example, in Pakistan, Afghanistan, Yemen, and Bangladesh, the EWP estimates are 0.936, 0.778, 0.43, and 0.427 higher than the AEWP, respectively, while in Saudi Arabia, Iran, and the Czech Republic, the EWP estimates are 0.204, 0.124, and 0.093 lower than the AEWP, respectively. Therefore, the EWP overestimates Pakistan, Afghanistan, Yemen and Bangladesh, while underestimating the development capacity of countries such as Saudi Arabia, Iran, and the Czech Republic. All these suggested that our revised method is effective, and the EWP after considering the planetary pressures is more reasonable.

All above data are technically validated and can be used for long-term monitoring and dynamic analysis of the regions. Perhaps more importantly, we produced relevant data for the new idea of sustainable development assessment. The data can be used separately for PHDI and AEWP assessment analysis or can be used to comprehensively evaluate according to the ideas provided in this paper.

### Limitations and future work

Although we present 29,621 data from 1990 to 2018 (part of them are to 2019) for 61 countries (including 11,154 raw data, 230 supplementary data and 18,237 pre-processing data calculated by ourselves), there are still several limitations of our provided database and need to be further improved in the future.

- The number of countries selected was limited due to serious missing data in many countries. Even in this paper, we tried our best to maximize the selection of 61 of the 65 B&R countries that were mostly mentioned in the literature and had to exclude 4 countries because of missing data. In the future, we will explore the data for more countries to construct a more comprehensive database of B&R, and even global.
- The period of the database was limited due to the different time span of different data that can be available. In order to make comprehensive use of data, the time span of the data selected must be consistent, and we can only choose the shortest time span according to the intersection of time spans. For example, the period of the database provided in this study can only be from 1990 to 2018, because the time span of the HDI available is from 1990 to 2019 and EF is from 1961 to 2018, although CDE is from 1960 to 2019 and MF is from 1970 to 2019. In the future, we should try to expand the data’s time span to provide longer time series data to promote more in-depth research. For example, we can calculate as much pre-1990 HDI data as possible according to the HDI calculation methodology.
- The database provided is based on the CPOE method we developed, a new idea for comprehensive evaluation on sustainable development by combining four composite indicators. In the future, we can work on a more comprehensive quantitative indicator based on the CPOE idea and produce the corresponding database, to promote sustainability research furtherly.

## Usage Notes

Research on evaluating sustainable development has long been a hot and challenging topic. Starting from the ultimate goal of sustainable development, this study is based on a new idea (CPOE) on comprehensive evaluation of sustainable development and provided a database with the B&R as a case. The database consists of 5 datasets, including 4 core datasets and 1 related dataset, covering 61 B&R countries, B&R regional average and global average from 1990 to 2018. All the generated data are annual and continuous, clear and easy to be understood, recorded as xlsx files and open to the public. They are readily read, updated and processed by many softwares, conducive to further exploration and development.

The database can be used to do the research and comparison of the temporal and spatial evolution dynamics of ecological consumption, planetary pressures, human well-being outputs, ecological well-being output efficiency alone, or comprehensive evaluation on sustainable development of the B&R. Its sharing and reusability are clear and broad, at least in three aspects.

First, the B&R covers different geographical units. The database can be used for the research and comparison between different geographical areas (such as arid and non-arid, tropical and non-tropical, high altitude and non-high altitude, etc.) and sub-areas (such as Asia and Europe, as well as Central Asia, West Asia, South Asia, the Middle East, Eastern Europe, and some biodiversity hotspots areas, etc.).

Second, the database also can be used to compare different socioeconomic areas (such as the six major economic corridors of the B&R, the Silk Road Economic Belt and the Maritime Silk Road, developed/developing/underdeveloped countries, etc.), regional organizations/cooperation forms (such as Association of Southeast Asian Nations, South Asian Association for Regional Cooperation, the Gulf Cooperation Council, Shanghai Cooperation Organization, and even BRICS and Asia-Pacific Economic Cooperation, etc.), and before and after the implementation of the BRI.

Third, the ideas and methods of this study can also be applied and extended to other non-B&R regions, and even the world, to gain more insights and further expand the database, and carry on the more in-depth research and objective demonstration of sustainable development assessment in national or sub-national, regional or sub-regional, and even global level.

## Supplementary information

Figure S3

Figure S4

Supplementary Information

Figure S1

Figure S2

## Data Availability

All figures of this study were generated by Origin (2022), all data were processed by using Microsoft Office Excel (Excel 2019), and the generated datasets have been stored as xlsx files and shared in Figshare^39^.
