# Supplementary figures and images for: Database of human well-being and eco-sustainability under planetary pressures of the Belt and Road 1990–2018
Source: Sci Data. 2023 May 20;10:309. doi: 10.1038/s41597-023-02231-x (PMC10199938; doi:10.1038/s41597-023-02231-x)

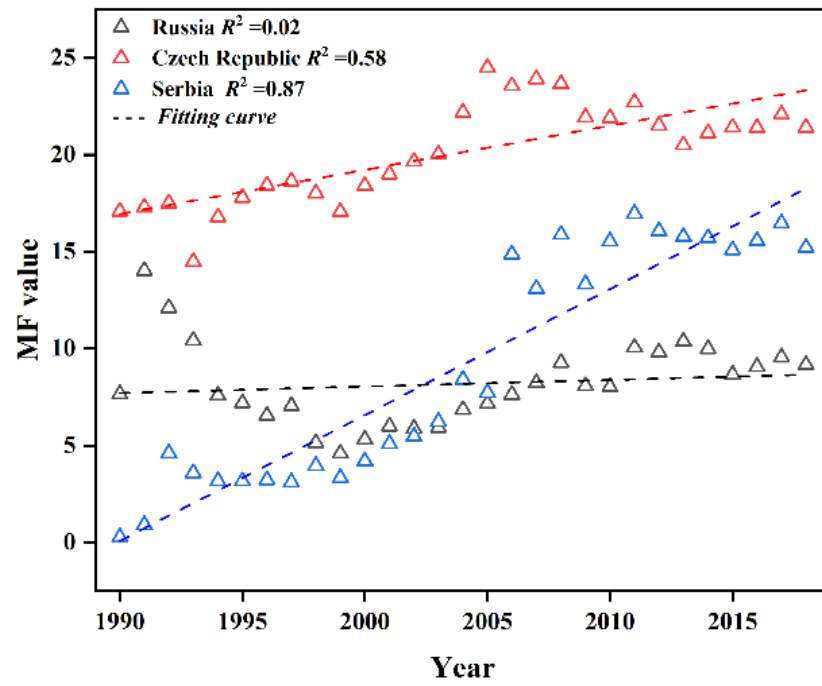

Supplement: Supplementary file 1 — Figure S3 [file 41597_2023_2231_MOESM1_ESM.pdf]

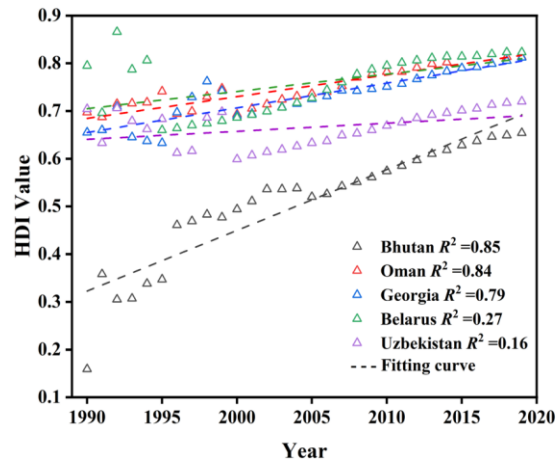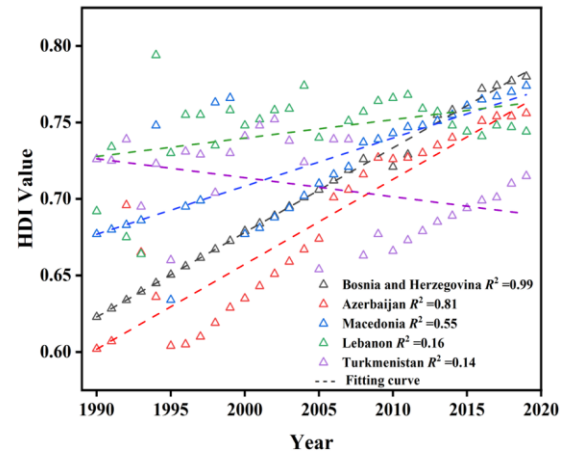

Supplement: Supplementary file 2 — Figure S4 [file 41597_2023_2231_MOESM2_ESM.pdf]

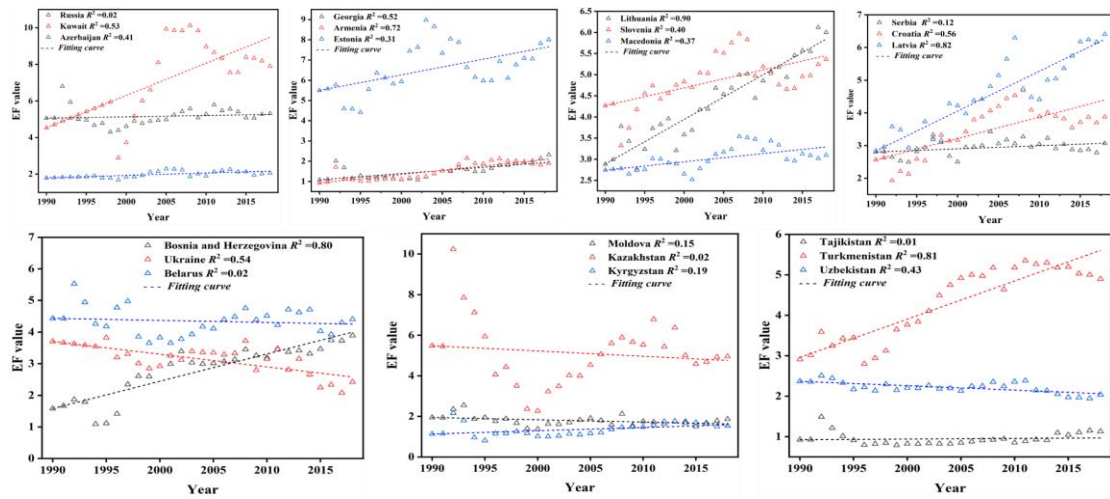

Supplement: Supplementary file 4 — Figure S1 [file 41597_2023_2231_MOESM4_ESM.pdf]

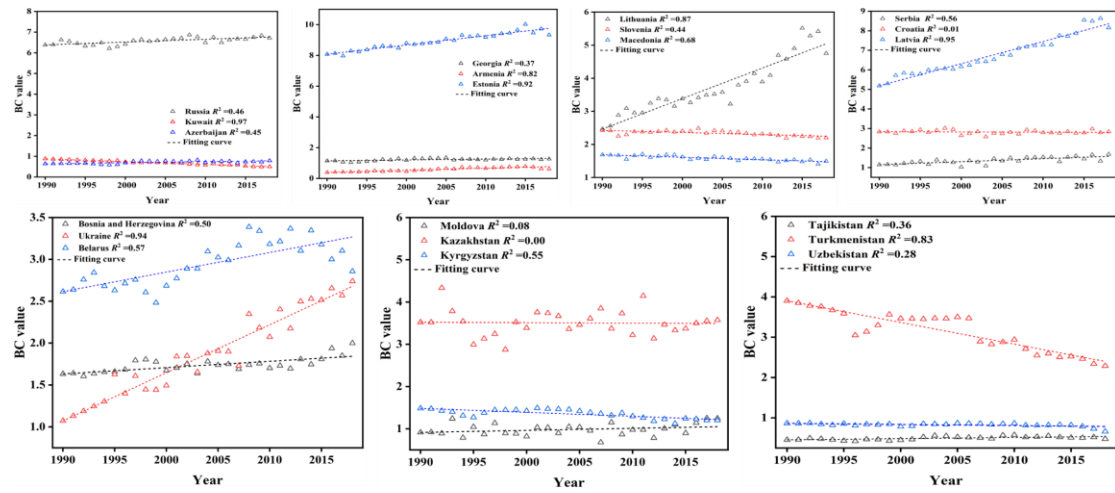

Supplement: Supplementary file 5 — Figure S2 [file 41597_2023_2231_MOESM5_ESM.pdf]
