# Supplementary material for: Database of human well-being and eco-sustainability under planetary pressures of the Belt and Road 1990–2018
Source: Sci Data. 2023 May 20;10:309. doi: 10.1038/s41597-023-02231-x (PMC10199938; doi:10.1038/s41597-023-02231-x)
Supplement: Supplementary file 3 — Supplementary Information [file 41597_2023_2231_MOESM3_ESM.pdf]

Table of contents

Supplementary Figure 1. Fitting results on the supplementary and raw data for ecological footprint (EF) per capita of 21 B&R countries from 1990-2018.

Supplementary Figure 2. Fitting results on the supplementary and raw data for biocapacity (BC) per capita of 21 B&R countries from 1990-2018.

Supplementary Figure 3. Fitting results on the supplementary and raw data for Material footprint (MF) per capita of 3 B&R countries from 1990-2018.

Supplementary Figure 4. Fitting results on the supplementary and raw data for Human Development Index (HDI) of 10 B&R countries from 1990-2019.

Supplementary Table 1. The list of the supplementary data.

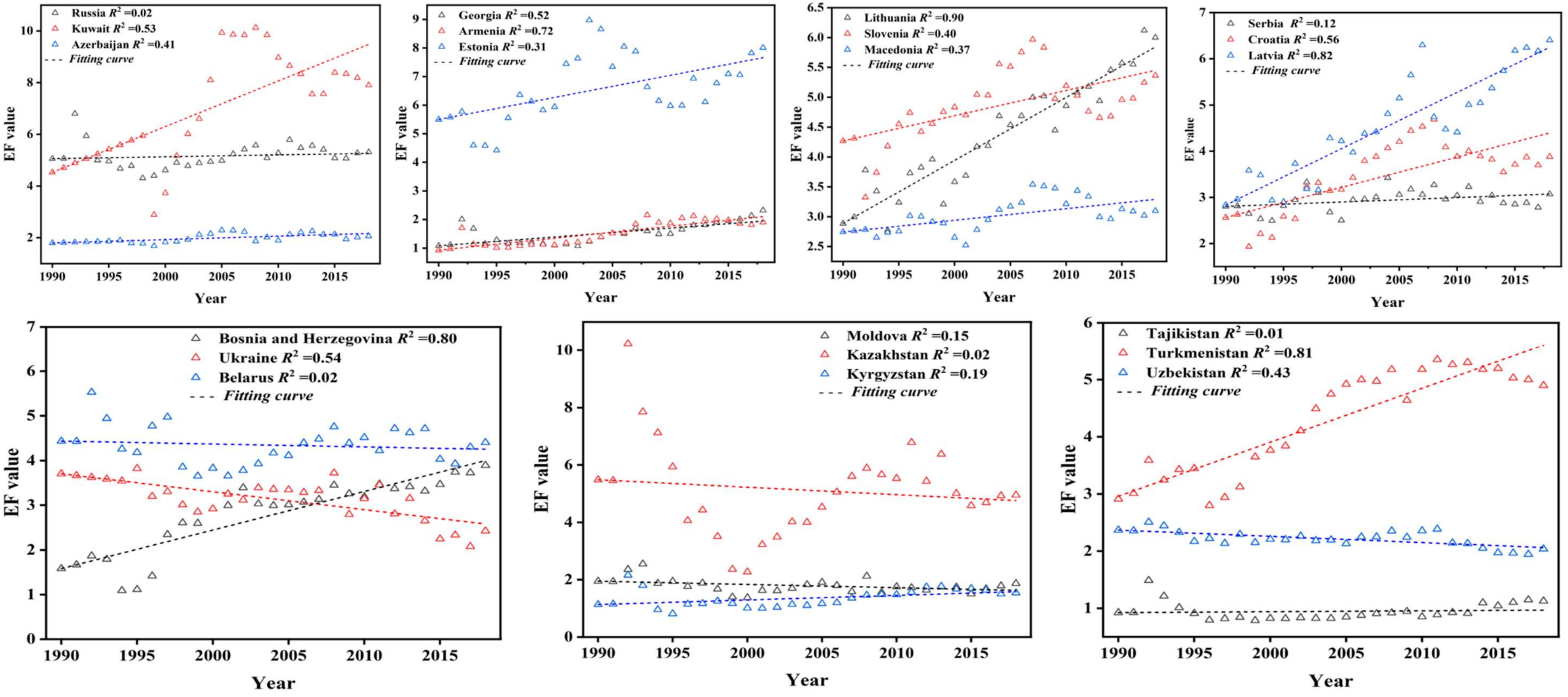

Figure S1. Fitting results on the supplementary and raw data for ecological footprint (EF) per capita of 21 B&R countries from 1990-2018.

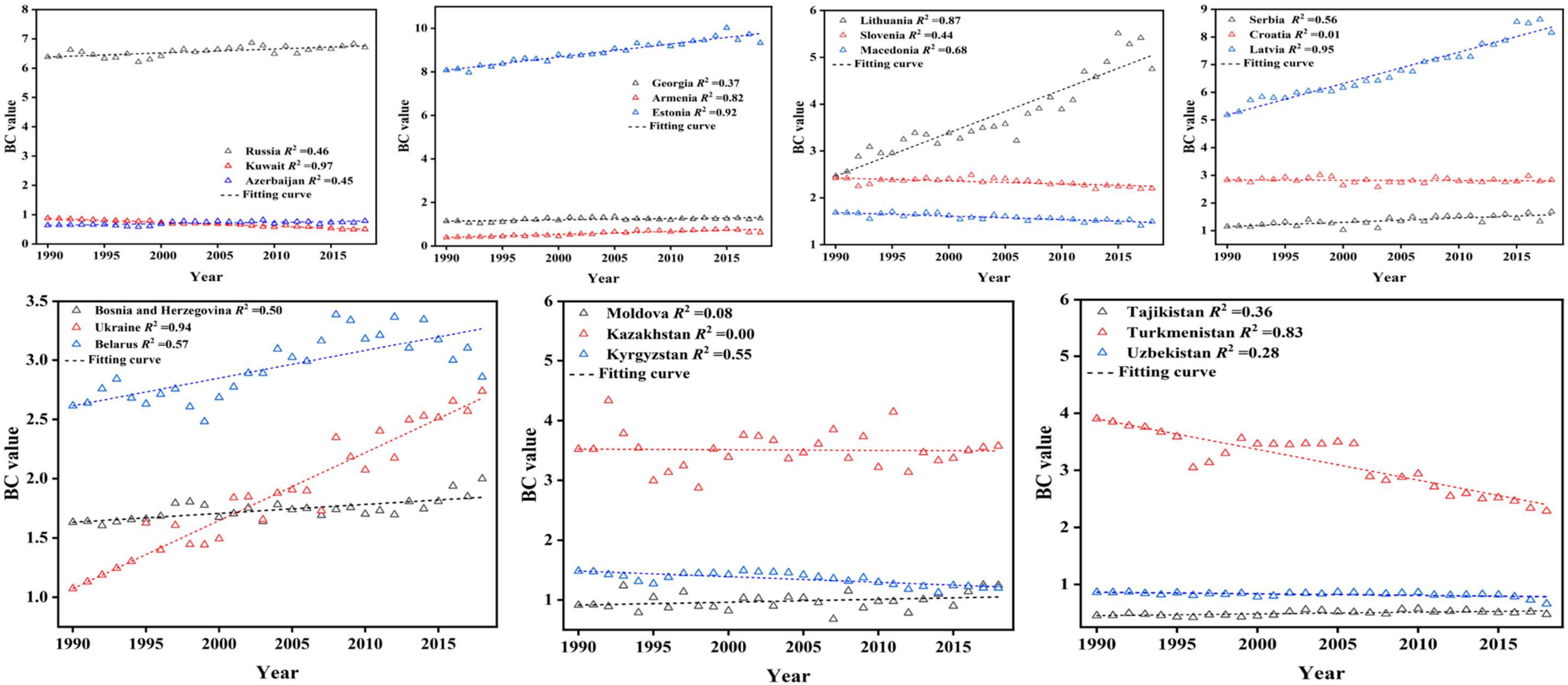

Figure S2. Fitting results on the supplementary and raw data for biocapacity (BC) per capita of 21 B&R countries from 1990-2018.

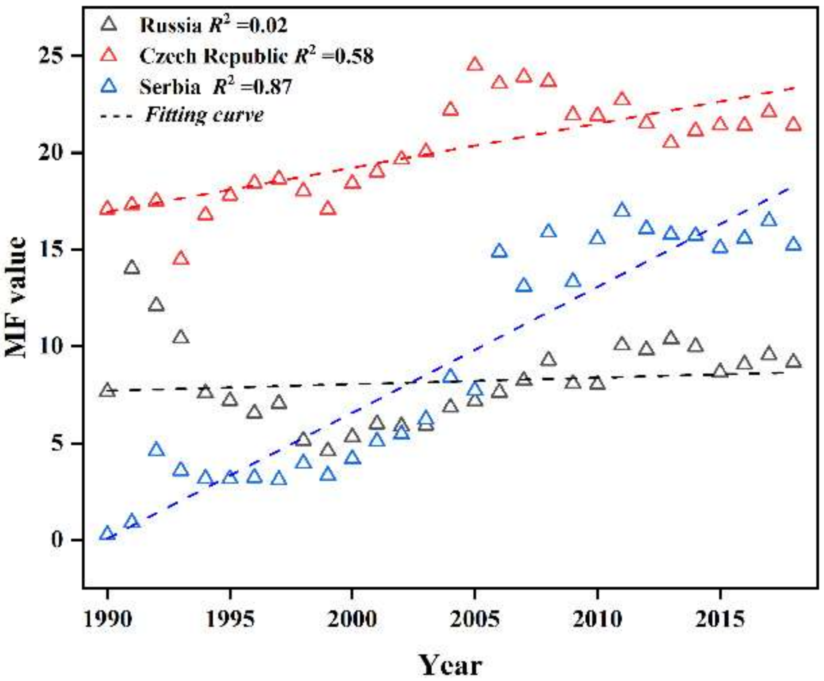

Figure S3. Fitting results on the supplementary and raw data for Material footprint (MF) per capita of 3 B&R countries from 1990-2018.

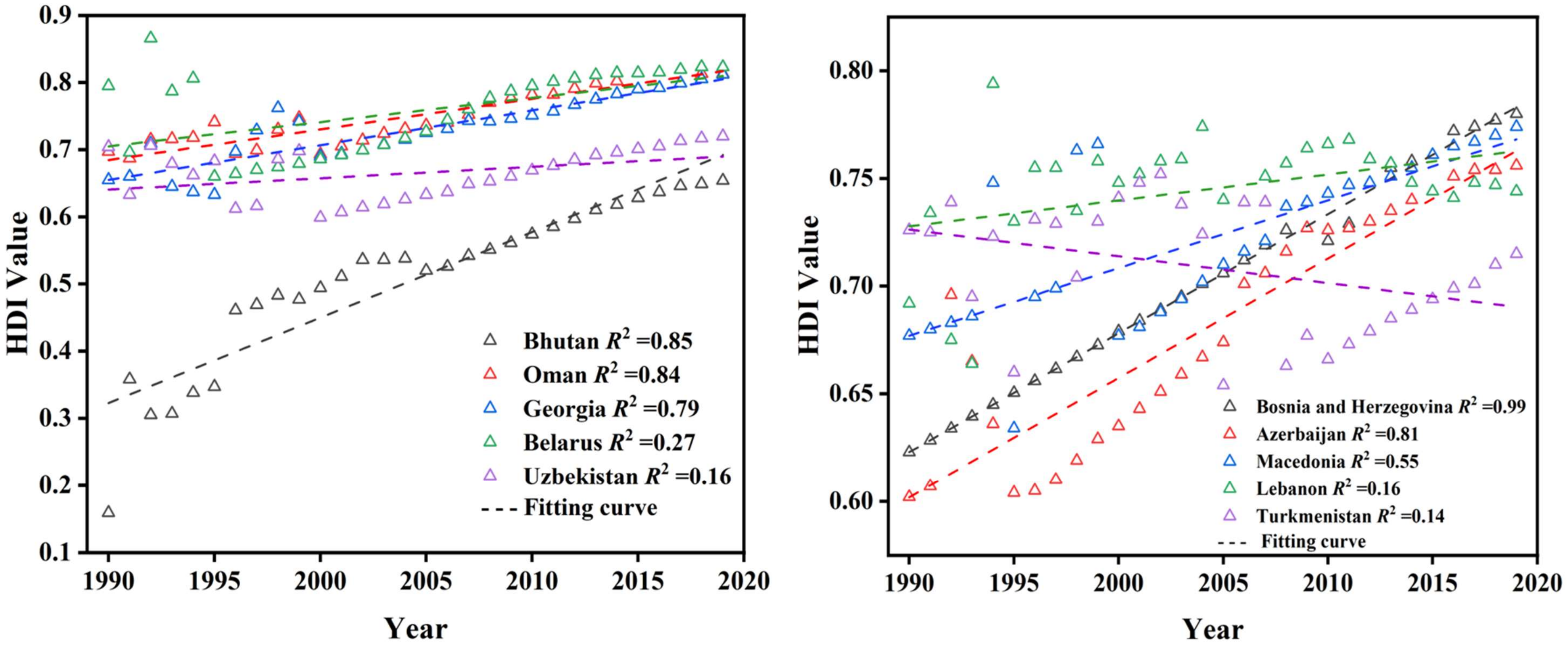

Figure S4. Fitting results on the supplementary and raw data for Human Development Index (HDI) of 10 B&R countries from 1990-2019.

Dataset types	Data types	Number of the raw data	Countries and years with missing data	Number of the supplementary data
**Consumption dataset**	Ecological Footprint (EF) per capita	1770	Russia (1990-1991), Kuwait (1990-1998), Azerbaijan (1990-1995), Georgia (1990-1991), Armenia (1990-1991), Estonia (1990-1991), Lithuania (1990-1991), Slovenia (1990-1991), Macedonia (1990-1992), Serbia (1990-1991), Croatia (1990-1991), Latvia (1990-1991), Bosnia and Herzegovina (1990-1991), Ukraine (1990-1994), Belarus (1990-1991), Moldova (1990-1991), Kazakhstan (1990-1991), Kyrgyzstan (1990-1991), Tajikistan (1990-1991), Turkmenistan (1990-1991), Uzbekistan (1990-1991).	57
**Pressure dataset**	Material footprint (MF) per capita	1854	Russia (1990), Czech Republic (1990-1992), Serbia (1990-1991).	6
**Output dataset**	Human Development Index (HDI)	1780	Bhutan (1990-2004), Oman (1990-1999), Lebanon (1990-2004), Azerbaijan (1990-1994), Georgia (1990-1999), Macedonia (1990-1999), Bosnia and Herzegovina (1990-1999), Belarus (1990-1994), Turkmenistan (1990-2009), Uzbekistan (1990-1999).	110
**Other dataset**	Biocapacity (BC) per capita	1770	Russia (1990-1991), Kuwait (1990-1998), Azerbaijan (1990-1995), Georgia (1990-1991), Armenia (1990-1991), Estonia (1990-1991), Lithuania (1990-1991), Slovenia (1990-1991), Macedonia (1990-1992), Serbia (1990-1991), Croatia (1990-1991), Latvia (1990-1991), Bosnia and Herzegovina (1990-1991), Ukraine (1990-1994), Belarus (1990-1991), Moldova (1990-1991), Kazakhstan (1990-1991), Kyrgyzstan (1990-1991), Tajikistan (1990-1991), Turkmenistan (1990-1991), Uzbekistan (1990-1991).	57
**Total**	—	7174	—	230

Table S1. The list of the supplementary data.
